# An assessment of microvascular hemodynamics in human macula

**DOI:** 10.1038/s41598-023-33490-8

**Published:** 2023-05-09

**Authors:** Dao-Yi Yu, Andrew Mehnert, Chandrakumar Balaratnasingam, Paula K. Yu, Martin Hein, Dong An, Stephen J. Cringle

**Affiliations:** 1 Centre for Ophthalmology and Visual Science, The University of Western Australia, Perth, Australia; 2 Lions Eye Institute, 2 Verdun St, Nedlands, Perth, WA 6009 Australia; 3 Sir Charles Gairdner Hospital, Perth, Australia

**Keywords:** Neuroscience, Physiology, Medical research

## Abstract

An adequate blood supply to meet the energy demands is essential for any tissue, particularly for high energy demand tissues such as the retina. A critical question is: How is the dynamic match between neuronal demands and blood supply achieved? We present a quantitative assessment of temporal and spatial variations in perfusion in the macular capillary network in 10 healthy human subjects using a non-invasive and label-free imaging technique. The assessment is based on the calculation of the coefficient of variation (CoV) of the perfusion signal from arterioles, venules and capillaries from a sequence of optical coherence tomography angiography images centred on the fovea. Significant heterogeneity of the spatial and temporal variation was found within arterioles, venules and capillary networks. The CoV values of the capillaries and smallest vessels were significantly higher than that in the larger vessels. Our results demonstrate the presence of significant heterogeneity of spatial and temporal variation within each element of the macular microvasculature, particularly in the capillaries and finer vessels. Our findings suggest that the dynamic match between neuronal demands and blood supply is achieved by frequent alteration of local blood flow evidenced by capillary perfusion variations both spatially and temporally in the macular region.

## Introduction

The central role of the cardiovascular system is to maintain an adequate capillary perfusion^1^. However, how this is achieved raises many unanswered questions. It has been evidenced that each cell in our body requires a substantial energy supply to survive and function^2^. An important question to be answered is: How is capillary perfusion regulated in order to provide enough support to meet the metabolic demands^3,4^? There is evidence that capillary perfusion is heterogeneous and is correlated with significant spatial and temporal variations corresponding to heterogeneity of local metabolic activity^5^. However, such perfusion properties have not been sufficiently explored and most information has been found experimentally from simple vascular systems such as the mesenteric^5^.

It is a challenge for capillaries to supply each cell in high energy demand neuronal tissues such as the brain and retina. The retina is reported to have the highest oxygen consumption per unit weight of any tissue in the human body^6–11^. The macula is recognized as the exquisitely specialized retinal region with high visual acuity and is supplied by retinal vasculature with a specific topographical distribution^12^. The eye is an optical organ which has evolved to limit the density of blood vessels in the retina, through which the light must pass to reach the photoreceptors. Therefore, it is expected that there are special challenges to ensure that the retina maintains an adequate blood supply. From previous in vivo studies of macular metabolism in monkeys, we demonstrated a high oxygen demand in the retina^11,13,14^ and even higher oxygen uptake in the macular compared to other areas of the retina^15^. The high-metabolic demands of the neurons and the relatively sparse nature of the retinal vasculature in the macular region are thought to contribute to the vulnerability of the macula to vascular disease^16^. An improved knowledge of macular capillary perfusion would improve the understanding of the pathogenesis of macular diseases.

Optical coherence tomography (OCT) and OCT angiography (OCTA) have been widely used clinically and provided an opportunity to investigate macular capillary perfusion^17–21^. The OCTA signal is derived from multiple OCT scans of the same retinal location and an evaluation of the changes in the backscattered OCT signal resulting from the movement of red blood cells (RBCs). This movement in turn reflects the changes of the regulation of blood vessels in response to cellular requirements in the various regions and can vary with time. The contrast in a single OCTA volume, or a 2D *en face* projection through the volume, thus visualizes that portion of the microvasculature in which RBC movement occurs over the course of the multiple OCT measurements. A sequence of OCTA images therefore records changes in the appearance/contrast of the microvasculature over time. Whilst the temporal resolution is clearly not sufficient to be able to quantify RBC flow, we have previously shown that the pixel-wise coefficient of variation (CoV) of OCTA intensity can be used to assess perfusion^18^. It enables the quantitative characterization of the variability in RBC movement in different portions of the network. For example, vessels that “blink” over the OCTA time series have a higher coefficient of variation than those that exhibit less pronounced intensity variation over time.

This study uses this CoV approach to explore the spatial and temporal variations of perfusion of the macular microvasculature in normal subjects to better understand the mystery of how a limited retinal blood supply can support such high neuronal demands and establish a baseline for normal perfusion. The latter may potentially be useful clinically to identify and characterize potential perfusion changes occurring in different stages of diabetic retinopathy and other retinal vascular diseases.

## Results

### CoV analysis results for each ROI category

**Figure 1:**
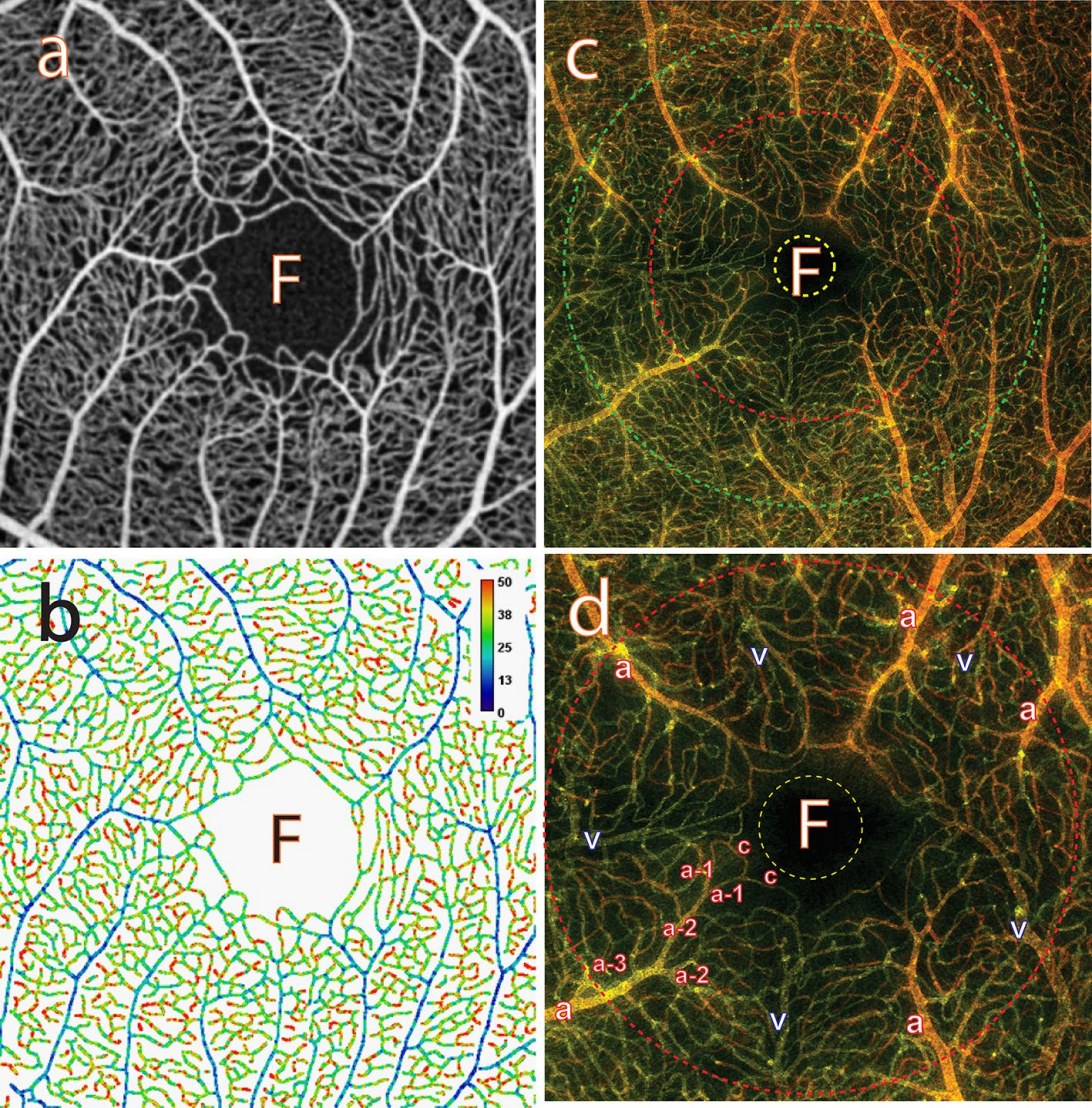
Use of knowledge of macular microvascular histology to evaluate the OCTA image. (**a**) Average of several aligned OCT angiograms (OCTA frames) from a normal subject. The FAZ is denoted “F” in (**a**), (**b**), (**c**) and (**d**). (**b**) Vessel centreline CoV map of the macular microvascular network from the same subject. The colour bar indicates CoV values capped at 50%, i.e., values greater than or equal to 50% have been assigned the red colour at the top of the colour bar. This has been done to accentuate the differences in CoV values (differences in colour). The visualisation shows that the major radial arterioles and venules appear blue in colour (indicating relatively low CoV) while the capillary network along the radial arterioles or venules including the tiny branches from arterioles and venules appear greenish to reddish (indicating high CoV). Interestingly there are some colour differences between each radial arteriole and venule and these colour differences are more obvious in some regions. (**c**) Confocal images (depth color-coded 2D projection through the image stack) of retinal microvasculature at the macular region obtained from a donor eye after perfusion staining labelled for filamentous actin. The foveola, fovea, and parafovea regions are indicated by three concentric circles (yellow, red and green respectively). Capillary density in the foveal region is less than that in the parafoveal region^27,28^. (**d**) A higher magnification image of the foveal region from (**c**) showing arterioles “a”, venules “v” and the subsequent branching of the retinal arteriole “a-3” into smaller arterioles “a-2” and “a-1” and capillaries “c”. Two capillaries “c” join to form a first order arteriole “a-1”, and two “a-1” arterioles join to form a second order arteriole “a-2”. The capillaries branching off the retinal arterioles are predominantly in the superficial half of the image stack (red pseudocolour) before connecting to the capillaries draining towards the retinal venules lying in the deeper half of the stack (green pseudocolour). Foveolar area is in the avascular region. Only some of the pairs of arterioles and venules enter the foveal region^27,28^.

**Figure 2:**
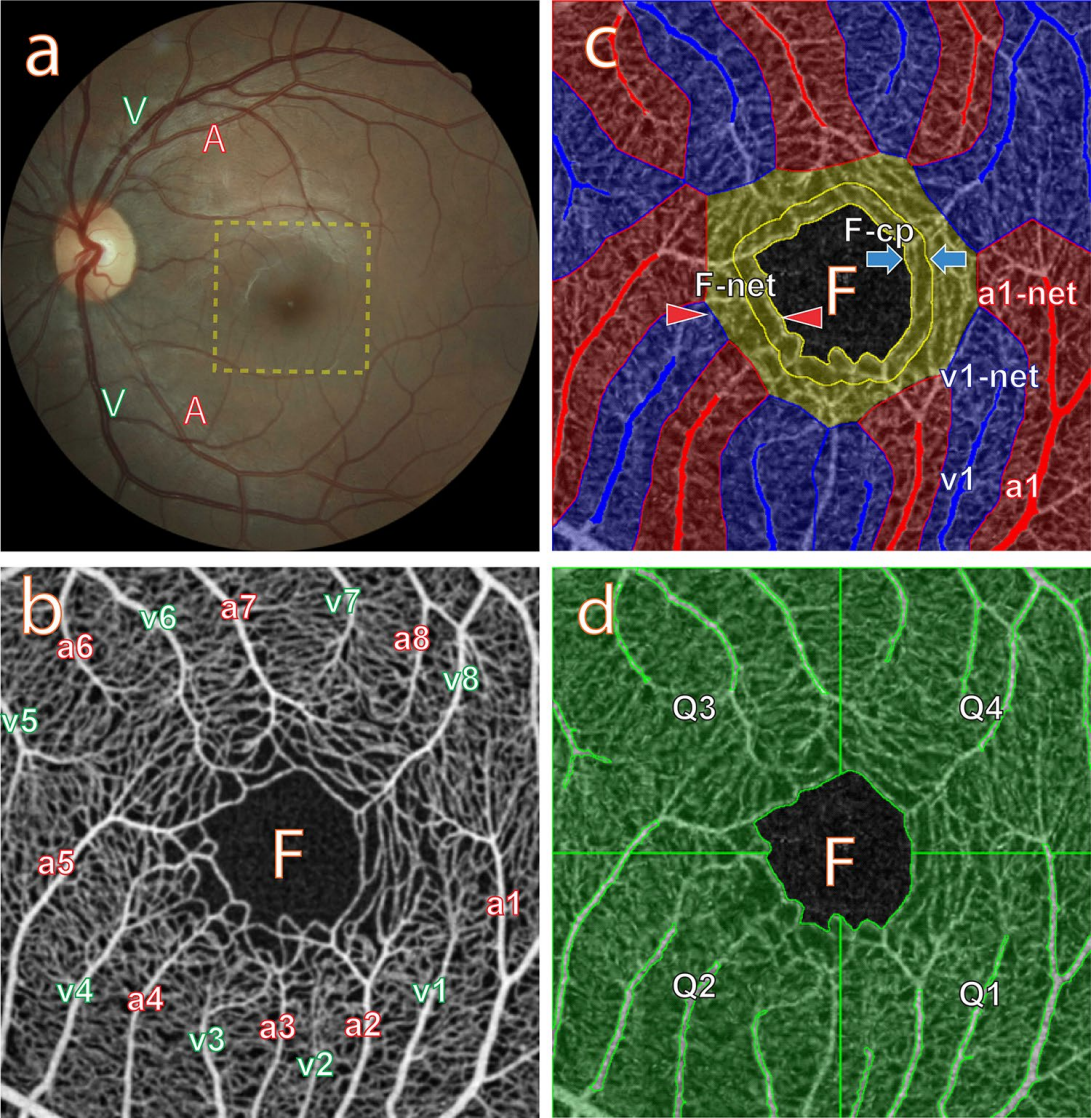
Macula and its microvasculature from a normal subject, illustrating the different ROI categories of macular microvasculature for the coefficient of variation study. (**a**) Colour fundus image: Retinal arteries “A” and veins “V” can be clearly identified. A dashed line box indicates the region sampled using OCTA imaging. (**b**) OCTA image (average of several aligned OCTA frames) of the macular area: There are eight pairs of radial arterioles “a” and venules “v”, the two being connected by a capillary network surrounding the foveola “F”. Only some of the arterioles enter the foveal region where they directly supply the terminal capillary ring. (**c**) First OCTA frame showing the selected major radial arteriole ROIs (“a1”–“a7” in solid red) and major radial venule ROIs (“v1”–“v8” in solid blue), the corresponding arteriole capillary network ROIs (“a1-net”–“a7-net” in outlined transparent red) and venule capillary network ROIs (“v1-net”–“v8-net” in outlined transparent blue), the ROI corresponding to the capillary network around the FAZ (“F-net” in transparent yellow) and the ROI corresponding to the capillaries around the foveal avascular zone (“F-cp” outlined in solid yellow lines). (**d**)First OCTA frame showing the capillary network quadrant ROIs (“Q1”–“Q4” in outlined transparent green).

**Figure 3:**
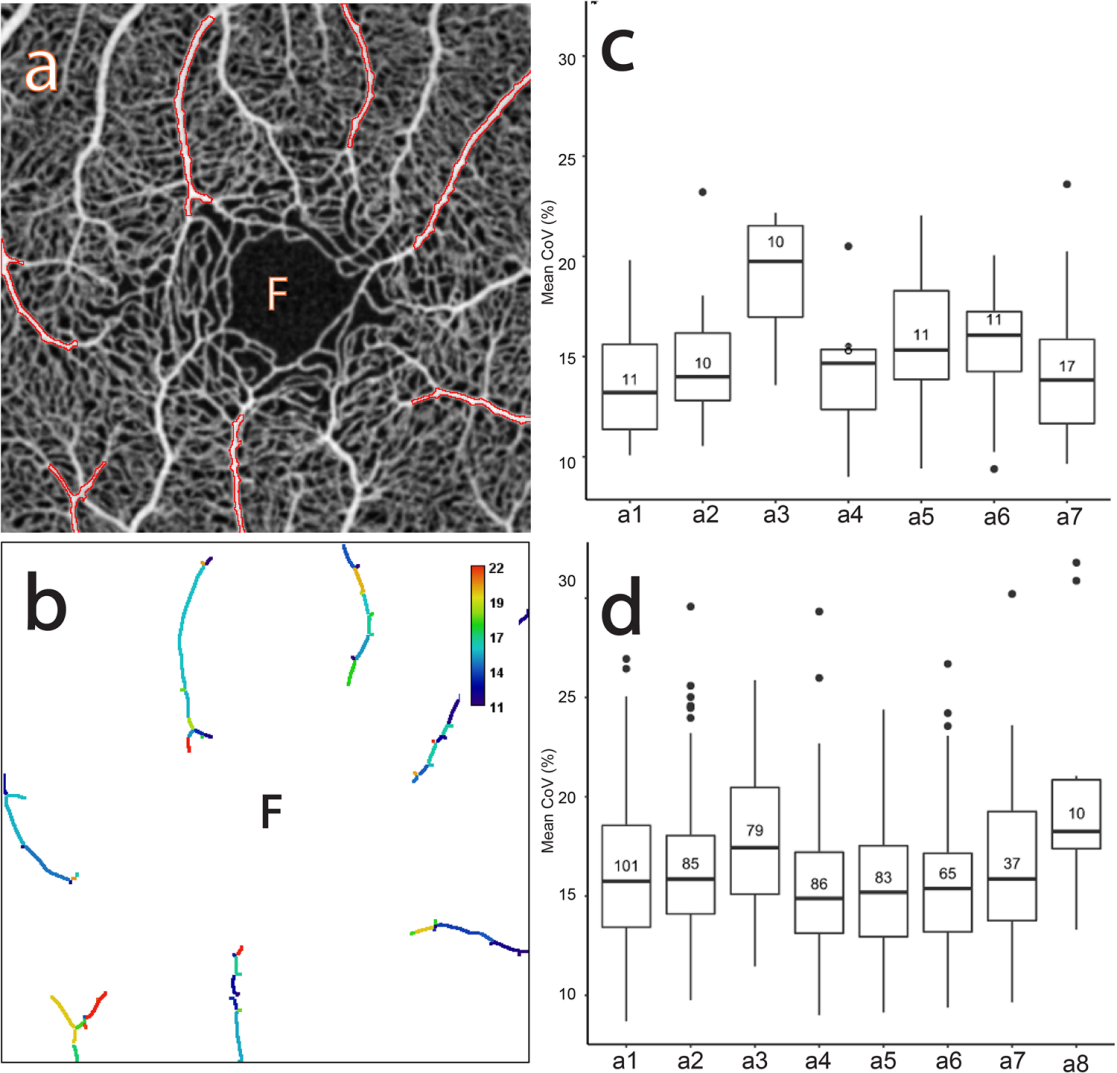
The CoV analysis of the major retinal arterioles in the macular capillary network. (**a**) Average of several aligned OCTA frames from the macular region of a normal subject showing selected major radial arteriole ROIs (“a1”–“a7” outlined in red). Note that each ROI includes order 2 to order 4 arterioles. The FAZ is denoted “F” in (**a**) and (**b**). (**b**) Corresponding mean CoV computed for individual vessel segments (sequences of pixels between junctions/end-points) within each arteriole ROI. Note that the CoV values in the arterioles are markedly lower than in the capillary network (see Fig. 1b) and unevenness of colour is found along each arteriole. Note also that the colour bar scale here is different to that in Fig. 1b and that CoV values have been clamped to the range shown (see text for details). (**c**) Comparative box plot of the mean CoV values computed for individual vessel segments for each ROI. The number in each box plot is the number of vessel segments. (**d**) Comparative box plot of the mean CoV values computed for individual vessel segments for each ROI pooled over 10 normal subjects.

**Figure 4:**
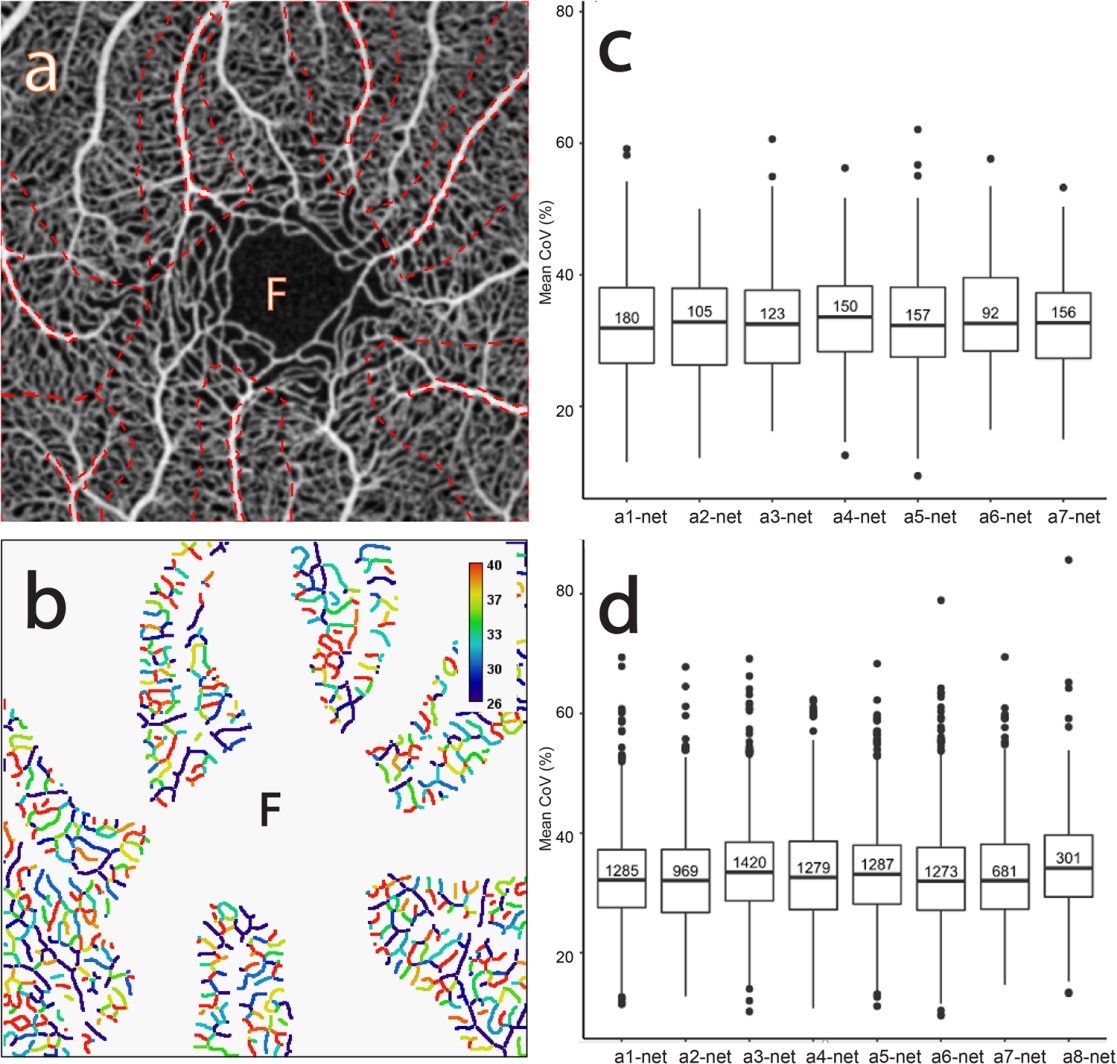
The CoV analysis of the capillary networks around the major radial arterioles. (**a**) Average of several aligned OCTA frames from the macular area of a normal subject showing the selected capillary networks around the major radial arteriole ROIs (“a1-net”–“a7-net” dashed outlined in red). (**b**) Corresponding mean CoV computed for individual vessel segments (sequences of pixels between junctions/end-points) within each capillary network ROI. Note that the CoV values in the capillary network are markedly higher than in the arterioles (see Fig. 1b). Note also that the colour bar scale here is different to that in Fig. 1b and that CoV values have been clamped to the range shown (see text for details). (**c**) Comparative box plot of the mean CoV values computed for individual vessel segments for each ROI. The number in each individual box plot is the number of vessel segments. (**d**) Comparative box plot of the mean CoV values computed for individual vessel segments for each ROI pooled over 10 normal subjects.

**Figure 5:**
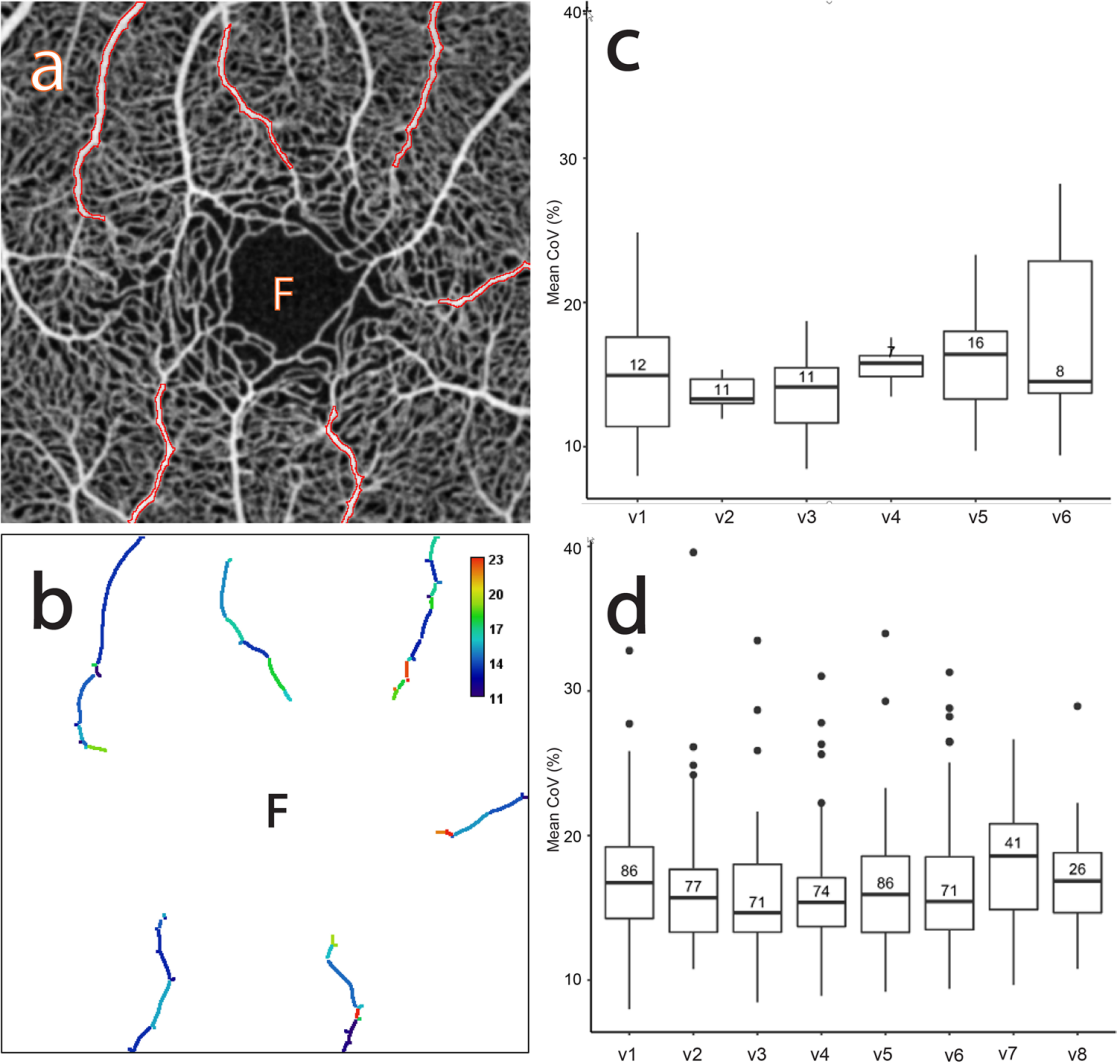
The CoV analysis of the major radial retinal venules. (**a**) Average of several aligned OCTA frames from the macular region of a normal subject showing selected major radial venule ROIs (“v1”–“v6” outlined in red). Note that each ROI includes order 2 to order 4 venules. (**b**) Corresponding mean CoV computed for individual vessel segments (sequences of pixels between junctions/end-points) within each venule ROI. Note that the CoV values in the venules are markedly lower than in the capillary network (see Fig. 1b) and unevenness of colour is found along each venule. Note also that the colour bar scale here is different to that in Fig. 1b and that CoV values have been clamped to the range shown (see text for details). (**c**) Comparative box plot of the mean CoV values computed for individual vessel segments for each ROI. The number in each individual box plot is the number of vessel segments. (**d**) Comparative box plot of the mean CoV values computed for individual vessel segments for each ROI pooled over 10 normal subjects.

**Figure 6:**
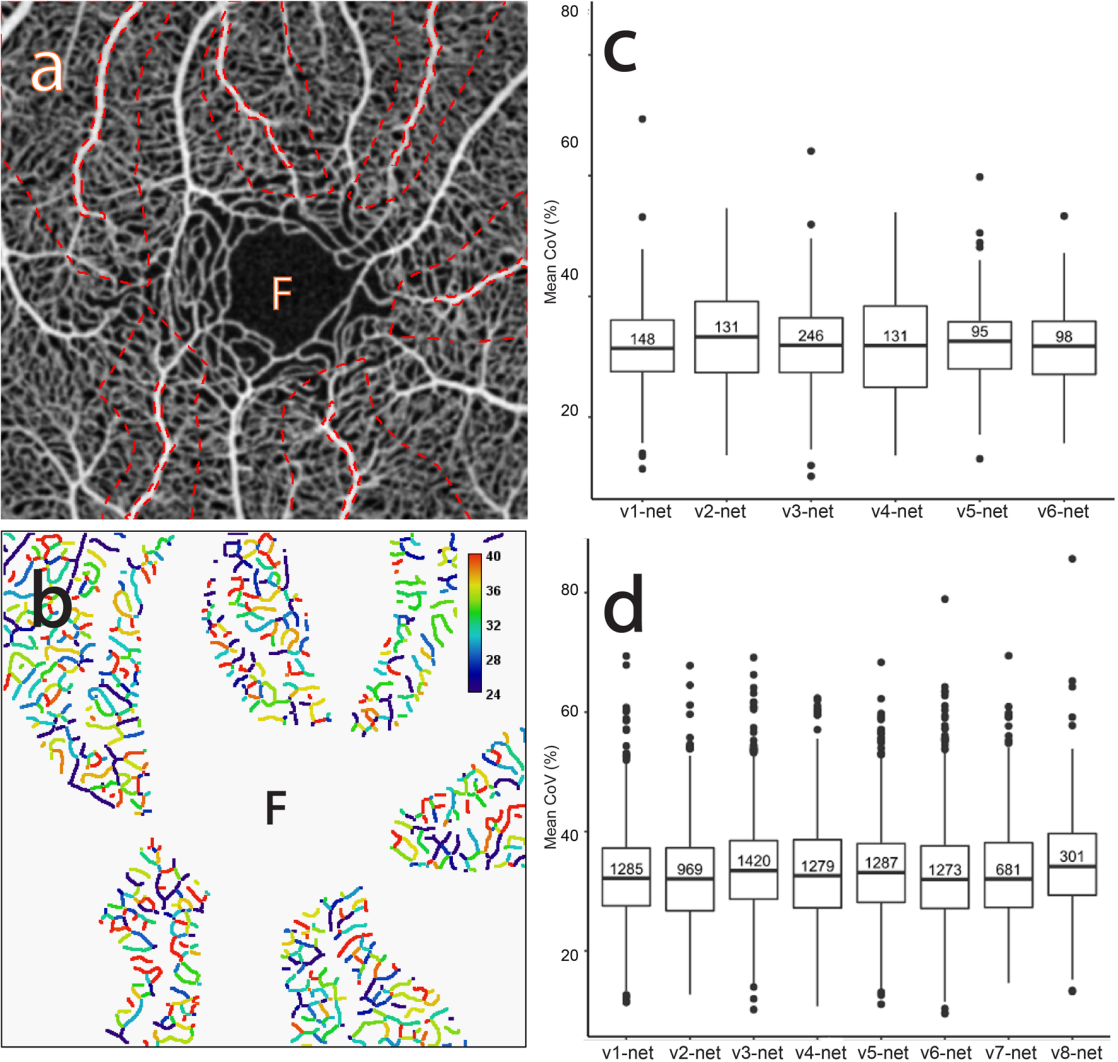
The CoV analysis of the capillary networks around the major retinal venules. (**a**) Average of several aligned OCTA frames from the macular region of a normal subject showing the selected capillary networks around the major radial venule ROIs (“v1-net”–“v6-net” dashed outlined in red). (**b**) Corresponding mean CoV computed for individual vessel segments (sequences of pixels between junctions/end-points) within each capillary network ROI. Note that the CoV values in the capillary network are markedly higher than in the venules (see Fig. 1b). Note also that the colour bar scale here is different to that in Fig. 1b and that CoV values have been clamped to the range shown (see text for details). (**c**) Comparative box plot of the mean CoV values computed for individual vessel segments for each ROI. The number in each individual box plot is the number of vessel segments. (**d**) Comparative box plot of the mean CoV values computed for individual vessel segments for each ROI pooled over 10 normal subjects.

**Figure 7:**
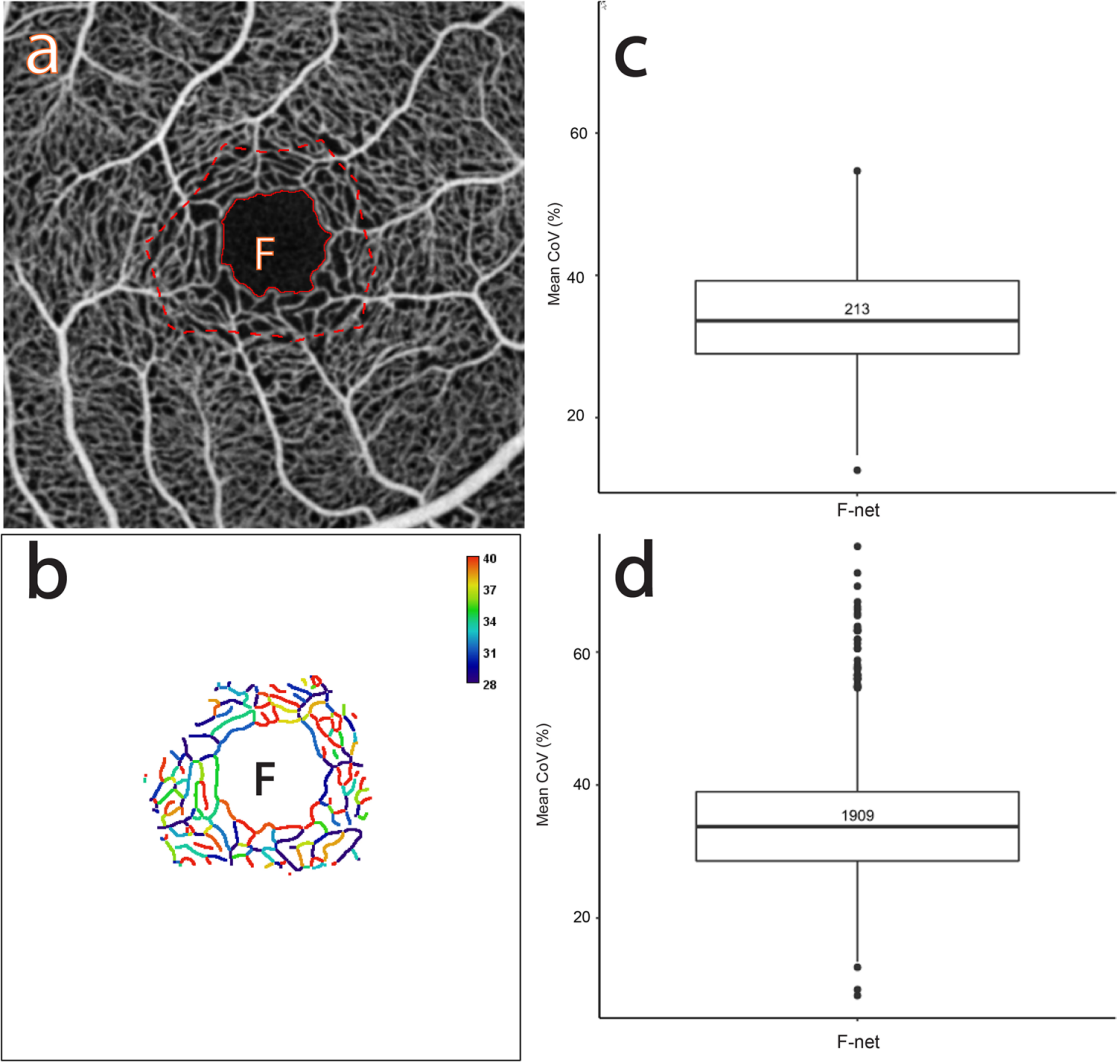
The CoV analysis of the capillary network around the foveal avascular zone. (**a**) Average of several aligned OCTA frames from the macular region of a normal subject showing the selected capillary network around the FAZ (“F-net” dashed outlined in red). (**b**) Corresponding mean CoV computed for individual vessel segments (sequences of pixels between junctions/end-points) within the ROI. Note that the CoV values in the capillary network are markedly higher than in the venules or arterioles (see Fig. 1b). Note also that the colour bar scale here is different to that in Fig. 1b and that CoV values have been clamped to the range shown (see text for details). (**c**) Box plot of the mean CoV values computed for individual vessel segments in the “F-net” ROI. The number in the box plot is the number of vessel segments. (**d**) Box plot of the mean CoV values computed for individual vessel segments in the “F-net” ROI pooled over 10 normal subjects.

**Figure 8:**
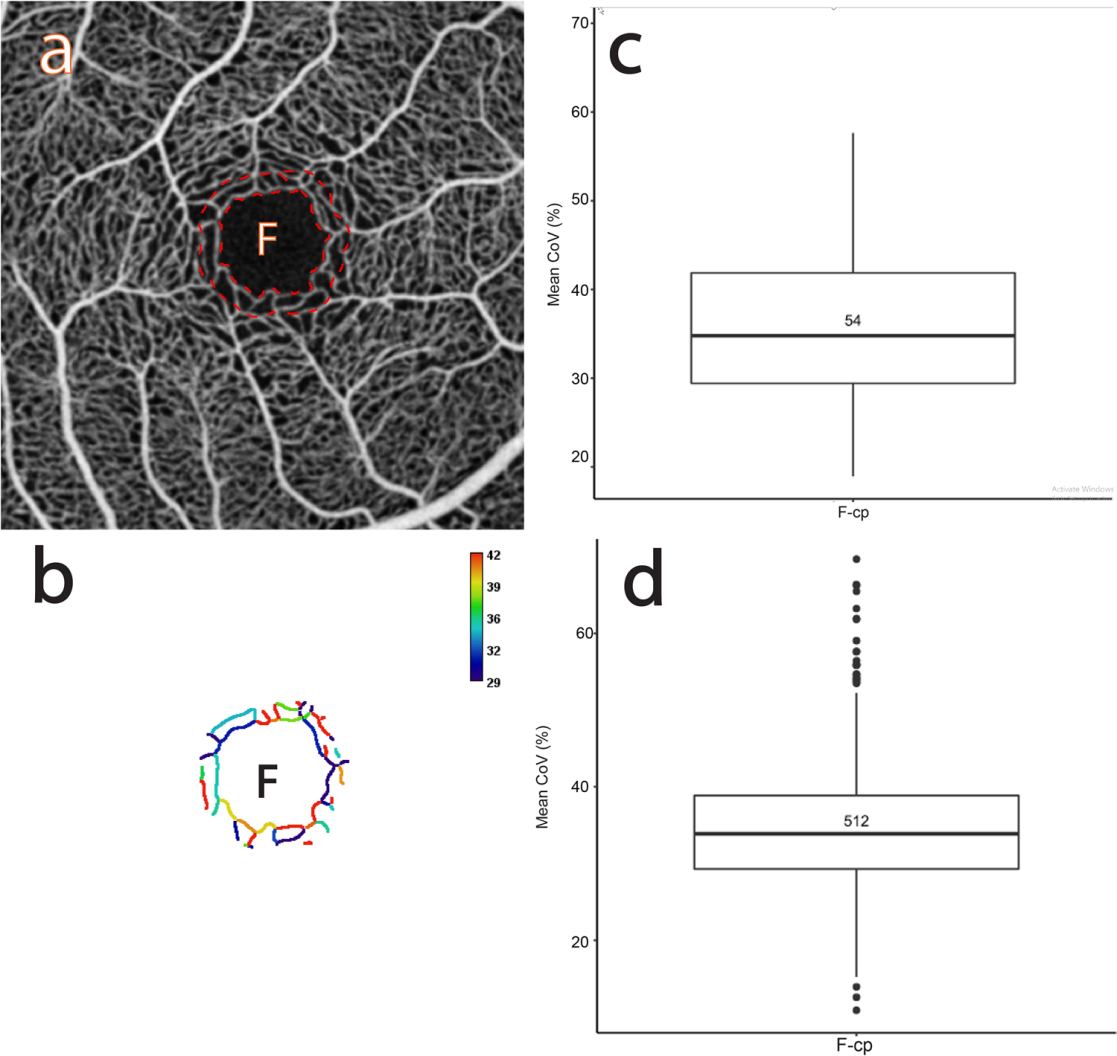
The CoV analysis of the capillaries around the foveal avascular zone. (**a**) Average of several aligned OCTA frames from the macular region of a normal subject showing the selected capillaries around the FAZ (“F-cp” dashed outlined in red). (**b**) Corresponding mean CoV computed for individual vessel segments (sequences of pixels between junctions/end-points) within the ROI. Note that the CoV values in the capillary network are markedly higher than in the venules or arterioles (see Fig. 1b). Note also that the colour bar scale here is different to that in Fig. 1b and that CoV values have been clamped to the range shown (see text for details). (**c**) Box plot of the mean CoV values computed for individual vessel segments in the “F-cp” ROI. The number in the box plot is the number of vessel segments. (**d**) Box plot of the mean CoV values computed for individual vessel segments in the “F-cp” ROI pooled over 10 normal subjects.

**Figure 9:**
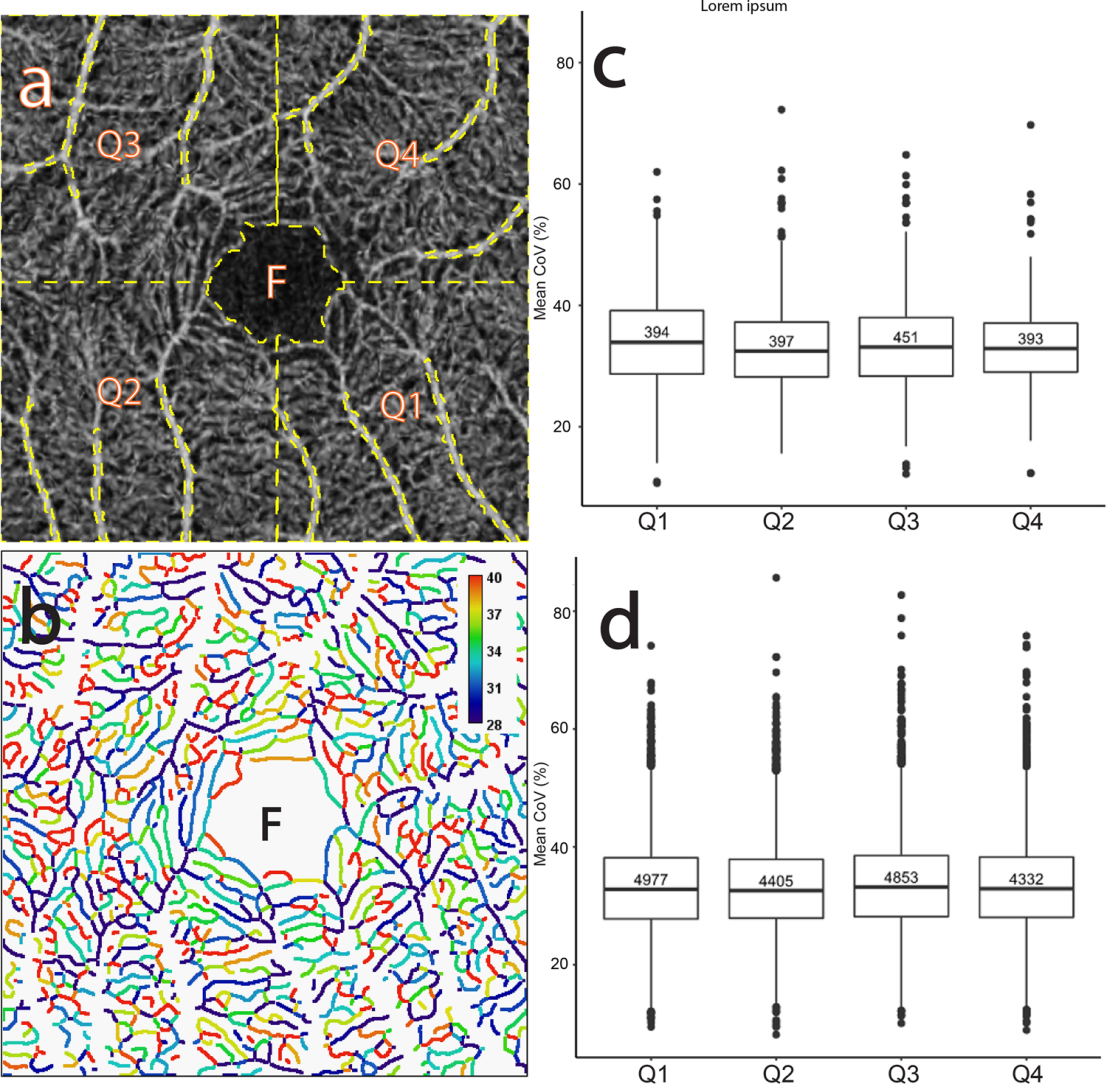
The CoV analysis of the capillary network quadrants in the macular area. (**a**) Average of several aligned OCTA frames from the macular region of a normal subject showing the selected capillary network quadrant ROIs (“Q1”–“Q4” dashed outlined in yellow). (**b**) Corresponding mean CoV computed for individual vessel segments (sequences of pixels between junctions/end-points) within each ROI. Note that the CoV values in these capillary network ROIs are markedly higher than in the venules and arterioles (see Fig. 1b). Note also that the colour bar scale here is different to that in Fig. 1b and that CoV values have been clamped to the range shown (see text for details). (**c**) Comparative box plot of the mean CoV values computed for individual vessel segments for each ROI. The number in each individual box plot is the number of vessel segments. (**d**) Comparative box plot of the mean CoV values computed for individual vessel segments for each ROI pooled over 10 normal subjects.

Figures 1 and 2 describe the CoV analysis approach and definitions of the region of interest (ROI) categories respectively (see "Materials and methods" for more detail). Figures 3, 4, 5, 6, 7, 8 and 9 show the CoV analysis results for one representative subject from each ROI category, as well as the results pooled over all 10 subjects. Sub-figure a shows the selected ROIs in the ROI category for the subject. Sub-figure b shows the corresponding vessel-segment mean CoV plot, visualising the spatio-temporal variation of each vessel segment. Here a vessel segment is defined to be a sequence of pixels between junctions/end-points in the single-pixel thick segmentation of the vessel centrelines (see "Materials and methods"). For each vessel-segment pixel, the CoV of its OCTA intensities over time has been computed. The mean of these CoV values is termed the vessel-segment mean CoV. This value has been used to colour-code each vessel segment. The colour bar shows vessel-segment mean CoV values ranging from least variation (dark blue) through to most variation (red). The values have been clamped to the range shown on the colour bar to accentuate differences in vessel-segment mean CoV value (differences in colour) between vessel segments (see "Materials and methods" for details). Sub-figure c shows the corresponding comparative box plot of vessel-segment mean CoV values for each of the ROIs. The median and interquartile range (IQR) for each ROI are measures of its temporal and spatial variation, respectively (these correspond to the thick horizontal black lines and box heights in the comparative box plots). Finally, sub-figure d shows the comparative box plot of vessel-segment mean CoV values for each of the ROIs pooled over all 10 subjects.

#### Major radial arterioles (“a1”, “a2”, …)

Figure 3a shows the selected major radial arteriole ROIs for one of the subjects. Figure 3b shows that there exist both local variations in the arterioles, indicative of temporal variation and along the arterioles, indicative of spatial variations.

Figure 3c shows the distribution of the vessel-segment mean CoV values for each ROI. The mean of the median values across all ROIs is 15.26% and the SD is 2.20% (both to two decimal places), whilst the mean of the IQR values across all ROIs is 3.83% and the SD is 0.68%. Figure 3d shows the distribution pooled over all 10 subjects. The mean of the median values is 16.07% and the SD is 1.17%, whilst the mean of the IQR values is 4.50% and the SD is 0.75%. This shows that the pooled data has less variability of temporal variation (smaller SD of the median values) but slightly higher variability of spatial variation (larger SD of the IQR values). In contrast, for the majority of subjects (data not shown) the pooled data has lower variability of both spatial variation and temporal variation across the ROIs.

#### Capillary network around the radial arterioles (“a1-net”, “a2-net, …)

Figure 4a shows the capillary network ROIs corresponding to the previously selected major radial arteriole ROIs shown in Fig. 3a. Figure 4b shows that the variation in each capillary network is larger than within the major radial arterioles. Numerous segments within each capillary network ROI show reddish or yellowish colours (relatively high CoV values) indicating high temporal variation in these segments, and where there are notable colour variations indicating high spatial variations are present. Although such temporal and spatial variations can also be found in the radial arterioles, they are much less pronounced.

Figure 4c shows the distribution of the vessel-segment mean CoV values for each ROI. The mean of the median values is 32.63% (SD = 0.52%) and the mean of the IQR values is 10.85% (SD = 0.69%), both of which are much higher than for the radial arterial ROIs. Figure 4d shows the distribution pooled over all 10 subjects. The mean of the median values is 32.80% (SD = 0.43%), whilst the mean of the IQR values is 10.60% (SD = 0.55%). The pooled data shows less variability (smaller SD values) across the ROIs. Indeed, this holds true for all the subjects in this study (data not shown).

#### Major radial venules (“v1”, “v2”, …)

Figure 5a shows the selected major radial venule ROIs for one of the subjects. Figure 5b shows that the intensity variation within the major radial venules is comparable to the major radial arterioles and is less than within the surrounding capillary networks. Nevertheless, like the arterioles the variation is not constant over time because there exist both local variations in the venules, indicative of temporal variation and along the venules, indicative of spatial variations.

Figure 5c shows the distribution of the vessel-segment mean CoV values for each ROI. The mean and standard deviation (SD) of the median values across all venule ROIs is 14.85% and 1.12% respectively (to two decimal places), whilst the mean of the IQR values is 4.50% and the SD is 2.90%. Figure 5d shows the distribution pooled over all 10 subjects. The mean of the median values is 16.16% and the SD is 1.21%, whilst the mean of the IQR values is 4.72% and the SD is 0.77% (these values are very close to those for the major arteriole ROIs). This shows that the pooled data has slightly more variability of temporal variation (larger SD of the median values) but less variability of spatial variation (smaller SD of the IQR values). In contrast, for most subjects the pooled data has lower variability of both spatial variation and temporal variation across the ROIs (data not shown).

#### Capillary network around the radial venules (“v1-net”, “v2-net, …)

Figure 6a shows the capillary network ROIs corresponding to the selected major radial venule ROIs shown in Fig. 5a. Figure 6b shows that the variation in each capillary network is larger than within the major radial venules. Numerous segments within each capillary network ROI show reddish or yellowish colours (relatively high CoV values) indicating high temporal variation in these segments, and where there are notable colour variations indicating high spatial variations are present. Although such temporal and spatial variations can also be found in the radial venules, they are much less pronounced.

Figure 6c shows the distribution of the vessel-segment mean CoV values for each ROI. The mean of the median values is 32.13% (SD = 0.68%) and the mean of the IQR values is 9.90% (SD = 2.21%). Figure 6d shows the distribution pooled over all 10 subjects. The mean of the median values is 32.70% (SD = 0.80%), whilst the mean of the IQR values is 10.36% (SD = 0.59%). This shows that the pooled data has slightly more variability of temporal variation (larger SD of the median values) but less variability of spatial variation (smaller SD of the IQR values). In contrast, for most subjects (data not shown) the pooled data has lower variability of both spatial variation and temporal variation across the ROIs.

#### Capillary network and small vessels around the FAZ

Figure 7a shows the “F-net” ROI for one of the subjects. The colours of the segments in Fig. 7b are very uneven with numerous reddish, yellowish, and bluish colour segments.

Figure 7c shows the distribution of the vessel-segment mean CoV for the “F-net” ROI. The median (measure of temporal variation) is 33.61% and the IQR (measure of spatial variation) is 10.26%. Interestingly, these values are very similar to the mean median and mean IQR for the capillary network ROIs around the major radial arterioles (“a1-net”, “a2-net”, …: 33.32% and 10.35% respectively) and for the capillary network ROIs around the major radial venules (“v1-net”, “v2-net”, …: 32.46% and 9.55% respectively) for the selected subject. Figure 7d shows the distribution pooled over all 10 subjects. The median is 33.75% and the IQR is 10.40%, which are very similar to the values for the selected subject and indeed for all subjects (medians ranging from 32.70%-35.80% and IQRs ranging from 8.72%-11.49%).

#### Band of pure capillaries around the FAZ

Figure 8a shows the “F-cp” ROI for one of the subjects. There is an unevenness of colours across the segments in Fig. 8b, with a colour distribution similar to the “F-net” ROI.

Figure 8c shows the distribution of the vessel-segment mean CoV for the “F-cp” ROI. The median (measure of temporal variation) is 34.80% and the IQR (measure of spatial variation) is 12.45%. Interestingly, these values are very similar to those of the “F-net” ROI (33.61% and 10.26% respectively). Figure 8d shows the distribution pooled over all 10 subjects. The median is 33.88% and the IQR is 9.59%, which are very similar to the values for the selected subject and indeed for all subjects (medians ranging from 31.26 to 37.27% and IQRs ranging from 7.61 to 12.45%).

#### Capillary network quadrants (“Q1”–“Q4”)

Figure 9a shows the division of the macular region into four quadrant ROIs (“Q1”–“Q4”) through the centre of the foveola. Each quadrant ROI excludes the major radial arteriole ROIs and venule ROIs and thus contains capillaries and tiny arterioles and venules. Figure 9b shows that each quadrant ROI contains ~ 500 segments and the colours of the segments are remarkably uneven with numerous reddish, yellowish, and bluish colour segments. The colour distribution is a combination of the distributions present in the capillary networks around the major radial arterioles, venules and the FAZ.

Figure 9c shows the distribution of the vessel-segment mean CoV for the ROIs. The mean of the median values across all quadrant ROIs is 33.10% (SD 0.61%) and the mean of the IQR values is 9.30% (SD is 1.00%). Figure 9d shows the distribution pooled over all 10 subjects. The mean of the median values is 32.87 (SD = 0.25%), whilst the mean of the IQR values is 10.23% (SD = 0.18%). This shows that the pooled data has less variability (smaller SD values) across the ROIs. Indeed, this holds true for all the subjects in this study (data not shown).

### Comparison of CoV between the seven ROI categories

**Figure 10:**
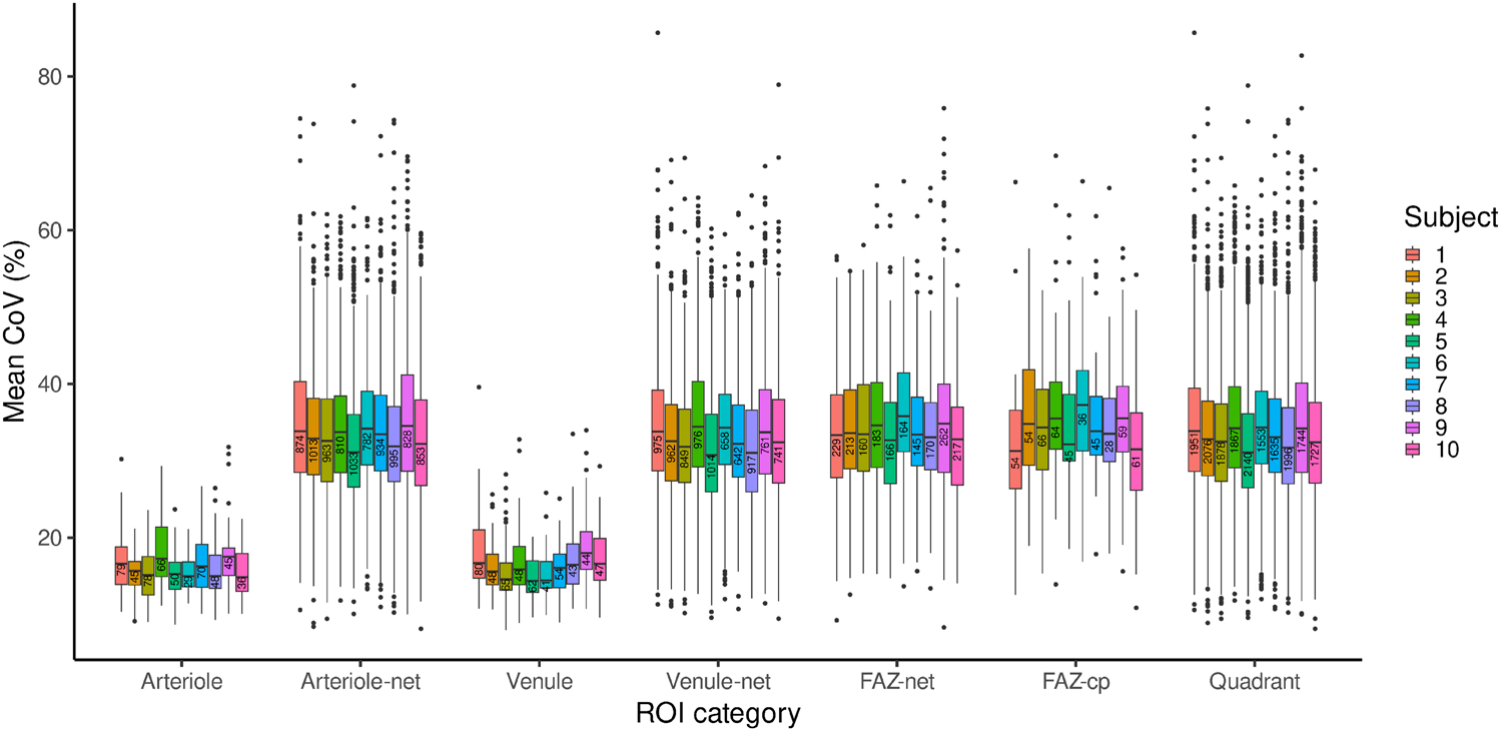
Vessel-segment mean coefficient of variation (CoV) for all 7 ROI categories across all subjects. The number within each box plot is the sample size. The category abbreviations are: “Arteriole” = major radial arteriole, “Arteriole-net” = capillary network around an arteriole, “Venule” = major radial venule, “Venule-net” = capillary network around a venule, “FAZ-net” = capillary network around the foveal avascular zone, “FAZ-cp” = pure capillaries around the foveal avascular zone, and “Quadrant” = capillary network quadrant.

Figure 10 shows a comparative box plot of the mean vessel segment CoV values pooled by category for each subject. The categories have been abbreviated as follows: “Arteriole” = major radial arteriole (containing “a1”, “a2”, …), “Arteriole-net” = capillary network around an arteriole (containing “a1-net”, “a2-net”, …), “Venule” = major radial venule (containing “v1”, “v2”, …), “Venule-net” = capillary network around a venule (containing “v1-net”, “v2-net”, …), “FAZ-net” = capillary network around the foveal avascular zone (containing “F-net”), “FAZ-cp” = pure capillaries around the foveal avascular zone (containing “F-cp”), and “Quadrant” = capillary network quadrant (containing “Q1”–“Q4”). Each box plot summarizes the distribution of mean CoV values for all vessel segments in a ROI category for a single subject. The number of vessel segments contributing to each box plot is shown inside below the median and can also be found in Supplementary Table S1 available online. It is immediately apparent that the categories fall into two groups: “Venule” and “Arteriole”, and all other (essentially capillary network) categories. The medians (measures of temporal variation) of the first group are much lower than those of the second group. Likewise, the IQRs (measures of spatial variation) of the first group are much smaller than the second.

**Figure 11:**
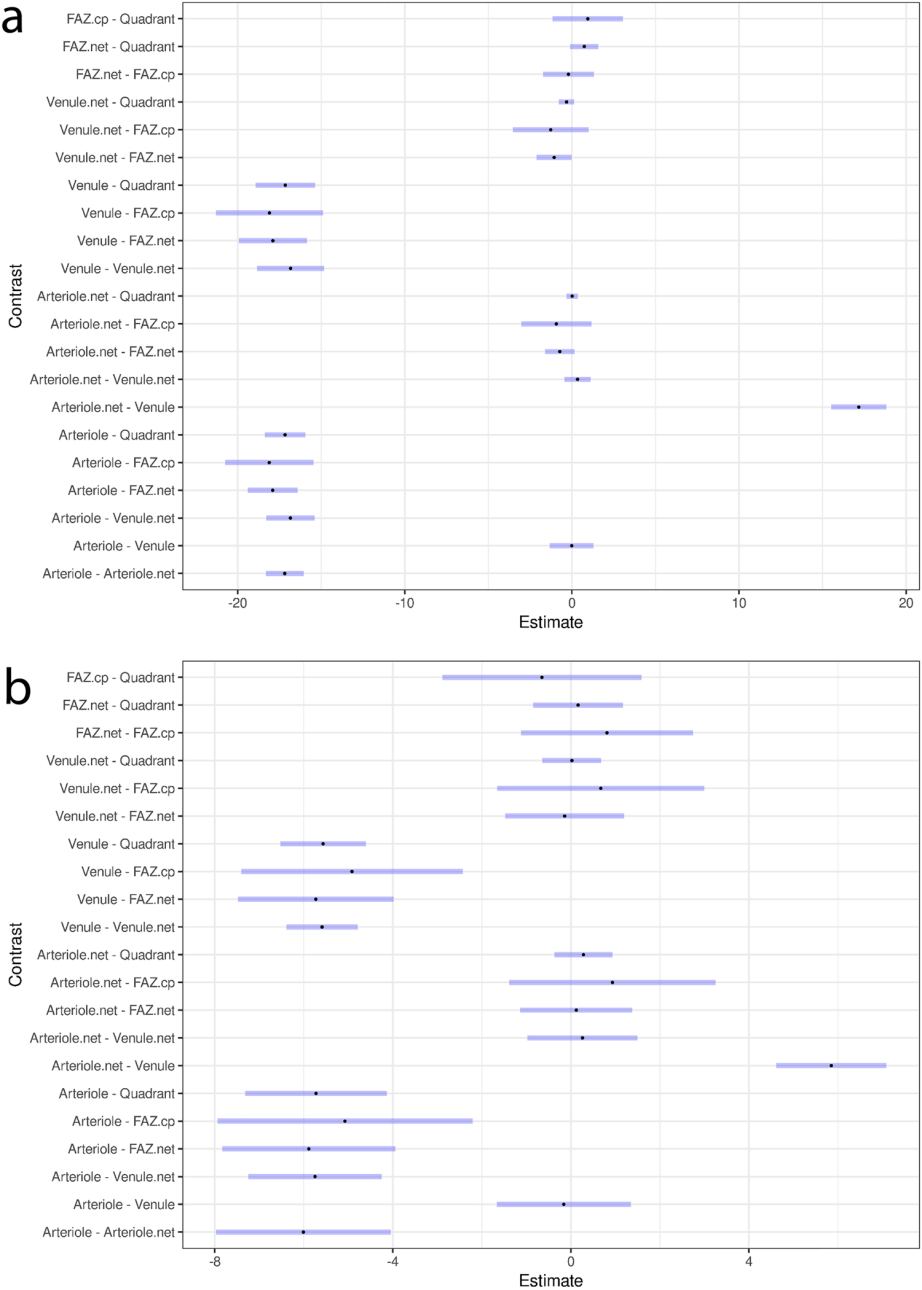
95% confidence intervals for the difference in medians (**a**) and IQRs (**b**) between each ROI category (see also Supplementary Tables S4 and S5 online). The CIs have been adjusted based on the Bonferroni method. The point estimate is shown as a dot and the confidence intervals are shown in blue.

The seven boxplot medians (one for each ROI category) for each subject in Fig. 10 were taken to be measurements under different conditions (see Supplementary Table S2 available online). A one-way repeated measures analysis of variance (RM ANOVA) was performed to test the null hypothesis that there is no difference between the medians (measures of temporal variation) between the different ROI categories. The data was checked to verify that it satisfied the test assumptions in relation to outliers and normality (Shapiro-Wilk’s test *p* > 0.05 for each ROI category). Mauchly’s test indicated that the assumption of sphericity had been violated (*p* < 0.001), therefore degrees of freedom were corrected using Huynh-Feldt estimates of sphericity (*ε* = 0.40)^22^. The result was significant, *F*(2.43, 21.86) = 888.29, *p* < 0.001, generalized *η*^2^ = 0.98. A post-hoc multiple comparison test, based on the paired-sample t-test, was performed to determine which ROI categories differ. Given the violation of sphericity, the Bonferroni method was chosen for the correction of *p* values^23^. Figure 11a shows 95% confidence intervals for these differences. The *p* values are shown in Table 1. The results suggest that there is no difference in temporal variation between the “Arteriole” and “Venule” categories and that these two categories are different to all the other categories. They also suggest that there is no difference between the remaining categories (essentially capillary networks) except between “Venule-net” and “FAZ-net” (only just significant).

**Table 1:** *p* values (to two decimal places) for the pairwise comparisons of ROI category medians (measures of temporal variation).

	Arteriole	Arteriole-net	Venule	Venule-net	FAZ-net	FAZ-cp
Arteriole-net	**0.00**					
Venule	1.00	**0.00**				
Venule-net	**0.00**	1.00	**0.00**			
FAZ-net	**0.00**	0.16	**0.00**	**0.05**		
FAZ-cp	**0.00**	1.00	**0.00**	0.93	1.00	
Quadrant	**0.00**	1.00	**0.00**	0.39	0.11	1.00

*p* values ≤ 5% level of significance have been indicated in bold.

Similarly, the seven boxplot IQRs (one for each ROI category) for each subject in Fig. 10 were taken to be measurements under different conditions (see Supplementary Table S3 available online) and a second RM ANOVA performed. The data was checked to verify that it satisfied the test assumptions in relation to outliers and normality (Shapiro-Wilk’s test *p* > 0.05 for each ROI category). Mauchly’s test indicated that the assumption of sphericity had been violated (*p* < 0.001), therefore degrees of freedom were corrected using Huynh-Feldt estimates of sphericity (*ε* = 0.64). The result was significant, *F*(3.83, 34.44) = 90.34, *p* = 0.00, generalized *η*^2^ = 0.88. A post-hoc multiple comparison test using Bonferroni correction was performed to determine which ROI categories differ. Figure 11b shows 95% confidence intervals for these differences. The *p* values are shown in Table 2. The results again suggest that there is no difference between the “Arteriole” and “Venule” categories and that these two categories are different to all the other categories. However, this time they also suggest that there is no difference between the remaining categories (essentially capillary networks).

**Table 2:** *p* values (to two decimal places) for the pairwise comparisons of ROI category IQRs (measures of spatial variation).

	Arteriole	Arteriole-net	Venule	Venule-net	FAZ-net	FAZ-cp
Arteriole-net	**0.00**					
Venule	1.00	**0.00**				
Venule-net	**0.00**	1.00	**0.00**			
FAZ-net	**0.00**	1.00	**0.00**	1.00		
FAZ-cp	**0.00**	1.00	**0.00**	1.00	1.00	
Quadrant	**0.00**	1.00	**0.00**	1.00	1.00	1.00

*p* values ≤ 5% level of significance have been indicated in bold.

## Discussion

### Major findings

The most pertinent findings in this study are: (1) spatial and temporal variation is present in each element of the macular microvasculature, (2) such variation can be quantified by CoV analysis, (3) CoV values in the capillaries and smallest vessels are significantly higher than that in the major radial arterioles and venules, and (4) there is no significant difference in CoV values in the capillaries of the four macula quadrants.

### Spatial and temporal variation and CoV analysis in the macular microvasculature

Adequate capillary perfusion is critical to supply each cell in our body^1^. The macular microvasculature distribution is complicated and sophisticated. We do not exactly know how it supplies highly packed neuronal cells with such limited blood flow. Our results clearly demonstrate that blood flow is not evenly and constantly distributed to individual capillaries as evidenced by the presence of spatial and temporal variation in each of the categories of the macular microvasculature, including radial arterioles and venules, capillary network and capillaries. The spatially and temporally heterogeneous nature of capillary perfusion is supported by some previous studies in other organs^5^. It has been thought to be correlated with significant heterogeneity of local oxygen consumption and metabolic activity^5^. Significant heterogeneity of intraretinal oxygen distribution and consumption, and oxygen modulation of neurovascular coupling in the retina, particularly in the macular area, have been demonstrated^9,15,24,25^. Therefore, we sought to test the hypothesis that there would be spatial and temporal variations of capillary perfusion in the macular microvasculature^18^. This study provides clear evidence from normal human subjects to support this hypothesis. Spatial and temporal variation of microvasculature has been also found in the brain. Arguably, our approach using CoV analysis of sequential OCTA images could provide an avenue to investigate the neurovascular coupling not only in the retina but also provide some useful information for brain studies.

By comparison of a sequential sequence of OCTA frames imaged from normal subjects, we were able to visualise the spatial and temporal variations in the macular microvasculature. However, it is a challenge to quantify the heterogeneous nature of such variations in the macular microvasculature. It is well known that different orders of vessels have various functional roles in the control of blood flow, particularly in the microvasculature^1,26^. We have previously described the orders of macular microvasculature in a human donor eye study using micro perfusion labelling techniques and retinal vascular histology to validate the OCTA technique^19,27–30^. In this study, we have used the knowledge based on vascular histology to divide the macular microvasculature into seven different categories to perform the CoV analysis. This approach allows us to study the CoV in each element of the macular microvasculature in normal subjects. It also could be used to discover possible changes in blood flow control in diabetic retinopathy and other retinal vascular diseases.

The CoV measure is not resistant to extreme values. Such values might occur because of noise or motion artefact. An alternative measure of dispersion that is less sensitive to extremes is the quartile coefficient of dispersion (QCD), which is defined in terms of the 1st and 3rd quartiles. We found that for the data exported from the Optovue system for this study, the pattern of variation was very similar between CoV and QCD (results not included in the paper). This suggests that the combination of denoising and motion artefact reduction in the Optovue system, and our non-linear alignment of OCT angiograms minimises the influence of outliers. In addition, the influence of outliers is further reduced because we compute statistics for sets of vessel segments, where each segment has been assigned the mean of the CoV values of its constituent pixels.

### CoV values in the macular microvasculature

Our results show that the CoV values in the capillaries and capillary networks is significantly higher than that in the major radial arterioles and venules. Our results suggest that such active regulation of blood flow occurs in the tiny micro vessels (less than order 2) rather than in larger radial vessels. In terms of precise locations of active regulation, it unlikely this could be determined from our findings. Higher CoV values in the capillaries evidenced by the terminal capillary ring and nearby capillaries around the foveola could be a consequence of active regulation of tiny vessels. While the capillary network around the radial vessels contains capillaries and small vessels less than order 2, it is not possible to identify these vessels in OCTA images. Whether capillaries have regulatory capability is controversial. It has been reported that capillaries do have regulatory capability^4,31^. However, other studies claim that it is the small arterioles that are the major site of control of capillary perfusion. The diameters of these small vessels are typically around 10 to 15 µm. The most important factor found thus far to affect the degree of opening and closing of these smallest arterioles is the oxygen tension in the tissues^32,33^. αSMA has been found in small retinal arterioles^34^. They are capable of being intermittently modulated resulting in temporal heterogeneity of capillary perfusion. Oxygen distribution in the retina is very significantly heterogeneous in each micro compartment^9,11,15,35^. Fluctuation in the rate of capillary perfusion and retinal tissue demands are often tightly interactive. Our findings are supported by a recent study in which spontaneous vasomotion along the cortical vascular tree has been reported using in vivo imaging techniques^36^. They have precisely identified the small arterioles (~ 10–20 µm) as the control site for neurovascular coupling and micro regional blood flow in mice.

Interestingly we have found that the CoV values could vary between each radial vessel and also between each capillary network. Such heterogeneity suggests that there are complicated control mechanisms and multiple control sites in the macular microvasculature. In addition to the complexity of topographic distribution of microvasculature, the vascular endothelial phenotype along the macular vascular tree is significantly heterogeneous^27,28^. Furthermore, to further study the control of capillary perfusion by small arterioles, αSMA labelling has been clearly found in some small arterioles, but not in others^34^. In fact, the interactions between neurons, glial cells and vessels are still one of the most challenging topics to be investigated.

We have found that there was no significant difference of capillary networks in CoV values between the four macula quadrants. This may provide some interesting information of the function of macular microvasculature. Despite the fact that blood flow through each capillary, capillary network or radial vessel varies in time, each capillary in the network can contribute to perfusion control. This information may provide useful clues for investigation of neurovascular coupling and identifying modulation strategies.

### Heterogeneity in capillary perfusion: an Important feature for macular microvasculature

There is a clear physiological basis which supports our finding that high heterogeneity in capillary perfusion exists in the macular microvasculature. Such high heterogeneity in capillary perfusion is to match high energy demands from the densely packed retinal neurons in the macular region. These neurons, their subcellular components and synapses are sophisticatedly arranged in the specific layers with unique topographic distribution resulting high heterogeneity of the energy demands. It has been demonstrated by intra retinal oxygen measurements. Significant differences of oxygen tension and high oxygen consumption in the distinct layers have been found from primate macular region by our and other groups^15,25^. Importantly, oxygen level in the inner retina is relatively low (~ 6 mmHg) close to critical oxygen level even in the normal condition. Furthermore, such heterogeneity can be alternated by different light stimulus^37,38^. Therefore, only strong and active local blood flow control within the capillary network can cope with such variations in metabolic demand spatially and temporally^18^. Due to the presence of fovea and foveola, macular microvasculature is uniquely arranged. Foveolar area is located in the avascular region. Only a few pairs of arterioles and venules enter the foveal region^27,28,39^. It is well known that the retinal vessels are lack of innervation and have more potent local regulatory capability^40,41^. When compared with that in other organs^1^. To match the neuronal demands, macular microvasculature could have some interesting features to perform active regulation the study on topographic distribution of contractile protein in the human macular region indicates the tiny arterioles could be active regulatory site^34^. These findings may provide some supportive information for high heterogeneity in capillary perfusion in the microvascular macula. However, up to now we still have limited knowledge to understand how macular microvasculature supplies each “hungry” neuron in the physiological and pathological conditions^3,4^.

## Materials and methods

### Subjects

This study was approved by the Human Research Ethics Committee of the University of Western Australia and has been carried out in accordance with The Code of Ethics of the World Medical Association (Declaration of Helsinki). Ten healthy subjects were recruited and one eye of each subject was randomly chosen to be imaged. Informed consent was obtained from all subjects. All subjects received ophthalmic examination and colour fundus photographs to exclude pre-existing ophthalmic conditions, including retinopathy and media opacity. All subjects reported no past medical history, and all had corrected visual acuity of 20/20 or better.

### OCTA image acquisition

OCTA images were acquired for each subject with the Optovue RTVue XR Avanti (Optovue, Inc., Freemont, CA, with AngioVue version 2018.1.0.43) which uses the split spectrum amplitude decorrelation angiography (SSADA) algorithm. This instrument has an A-scan rate of 70,000 scans per second using a light source centred on 840 nm and a bandwidth of 45 nm. Each OCTA volume contains 304 × 304 A-scans with two consecutive B-scans captured at each fixed position before proceeding to the next sampling location. The total acquisition time for a single volume was approximately 3 s. The system employs two strategies to reduce motion artefacts arising from eye blinks, saccades, and fixation changes. The first is real-time eye tracking, permitting the reacquisition of a portion of the OCTA volume when motion is detected. The second is to acquire a pair of consecutive OCTA volumes with B-scans orthogonal to one another, and to spatially co-register and combine the two volumes as a software post-processing step. The instrument software automatically segments the different retinal boundaries in the merged volume and generates a 2D *en face* projection through the retinal thickness being evaluated. The resulting OCT angiogram can then be exported as a raw file.

The scan area was 3 × 3 mm centred on the fovea. A final (merged) volume was accepted if the Scan Quality score was 8 or better, and the volume was free from major motion artefacts evidenced by vertical or horizontal lines in the image. A total of 20 such volumes were acquired for each subject. Our preliminary data has shown that spatio-temporal variations in capillary perfusion can be reliably detected using this number of angiograms^18^. For each volume, the OCT angiogram for the full retinal slab was exported as a raw file.

### Image analysis

CoV is a dimensionless measure of the spread or dispersion of data about its mean, defined as the standard deviation divided by the mean and generally expressed as a percentage. It was selected as a quantitative measure of pixel-wise signal intensity variation in a sequence of OCT angiograms for the following reasons. First, it is zero when there is no variation over time. Second, it is independent of the ordering of the observations. This reflects the fact we do not characterize RBC movement in terms of a rate or direction of change over time, but only in terms of variation relative to the mean. Third, it is independent of linear scaling. This means it is invariant to the presence of a bias field (i.e., where the imaging system yields different intensity scaling at different spatial locations where the reflectance properties should be identical). Finally, it permits the comparison of the variation from one data series to another even where the means are very different. This means we can compare different retinal locations within an eye, between a subject’s eyes, and between the eyes of several subjects.

Custom software was written for FIJI^42^ and R^43^ to quantitatively characterise the spatio-temporal intensity variation in a sequence of OCT angiograms, hereinafter called OCTA frames, in terms of pixel-wise CoV.

The software performs the following steps:

1. Upscaling of each frame by a factor of two (bilinear interpolation) to facilitate step 4
2. Spatial alignment of the frames using rigid followed by non-linear registration ^44^
3. Computation of the CoV pixel-wise to produce a CoV map
4. Segmentation (i.e., detection and delineation) of single-pixel thick vessel centrelines
5. Computation of summary CoV statistics for vessel segments (sequences of pixels between junctions/end-points in the centreline image from step 4) in selected regions of interest (ROIs).

The set of CoV values along the vessel centrelines (step 4) constitutes the centreline CoV map. This map can be visualised using a false colour rendering (e.g., a heat map from dark blue through to red as in Fig. 1b). Individual CoV values in the map provide information about temporal intensity variation and sets of CoV values along centrelines provide information about spatial variation.

### Definition of ROI categories for the optimum analysis of the macular microvasculature based on knowledge of macular microvascular histology

Figure 1 is to explain the optimum approach to perform CoV analysis of the macular microvasculature from OCTA images. Figure 1a is the average of aligned OCTA frames from a normal subject. The changes in microvasculature intensity between consecutive frames occur both spatially (different locations) and temporally (different frames taken at various time points). This can be seen in Fig. 1b, which is the vessel centreline CoV map for the same sequence of OCTA frames. The colour bar shows a range of 0 to 50%. All CoV values greater than 50% have been capped at 50% to accentuate the differences in CoV values (differences in colour) within and between the radial arterioles and venules, and the capillary network. There is a dramatic difference between the radial arterioles and venules (blueish colour, low CoV values) and the capillaries and fine vessels (greenish, yellowish and reddish colours indicating higher CoV values).

Whilst current OCTA yields retinal microvascular images down to the capillary level, it cannot reliably differentiate between capillaries and tiny arterioles or venules because it does not provide any cellular information about vessel wall properties. The optimum way to study spatio-temporal variation of perfusion in the arterioles, venules and capillaries is to leverage knowledge gained from vascular histology. By taking advantage of identifiable topological correspondences in microvasculature in the macular area, we can transfer knowledge gained from histology of a human donor eye to OCTA images. Figure 1c is a representative confocal image (depth color-coded 2D projection through the image stack) of the retinal microvasculature in the macular area from a human donor eye (not the same subject as in Fig. 1a). The macular microvasculature, including endothelial cells and smooth muscle cells, was labelled using arterial perfusion staining^27^. The topology of the vascular trees can be described using centripetal Horton-Strahler (H-S) ordering and the centrifugal generation scheme (CGS)^1^ The H-S approach starts at the capillary level and proceeds centripetally. The order is increased if two segments of equal order join at a bifurcation. The CGS starts from the most central vessel considered and proceeds to the capillary level, increasing the generation by one at every branch point. Figure 1d is a higher magnification of the foveal region showing arterioles, venules and the subsequent branching of the retinal arteriole into smaller arterioles “a-2” and “a-1”, and capillaries “c”. Following the H-S approach, the two capillaries “c” join to form a first order arteriole “a-1”, and two “a-1” arterioles join to form a second order arteriole “a-2” and then to the major arteriole “a-3”. The venules “v-1”, “v-2”, “v-3” and “v-4” are similarly identified. The topological features of the macular microvasculature seen in OCTA images are comparable with those seen in fluorescein images and vascular histology^19^. The terminal capillary ring with some nearby anastomosed capillaries can be identified from OCTA images (Fig. 1a) based on knowledge from vascular histology (Fig. 1c,d). The capillary network between a paired radial arteriole and radial venule contains the capillaries and tiny branches of the arteriole and venule (“a-1” or “v-1”). It is evident that there are many bifurcations from each radial arteriole or venule indicating that many generations are present along a short segment length. Some arterioles and venules only reach the parafoveal or perifoveal region. Higher order arterioles sprout smaller branches of capillaries and arterioles which further bifurcate to form the capillary networks before converging to the venules. The number of vessel generations in the macular area imaged is ~ 12 and there are ~ 4 different orders of vessel. This then motivates the definition of ROI categories that can be reliably studied using OCTA. The colour fundus image (Fig. 2a) is used to identify each radial arteriole and venule in the matching mean projection OCTA image (Fig. 2b). In particular, we start by selecting vessels of order 2 to order 4 to define ROIs corresponding to radial arterioles and radial venules. We then define capillary ROIs by partitioning the capillary network with respect to the set of arterioles, venules and the foveal avascular zone (FAZ) in terms of Euclidean influence zones (generalised Voronoi polygons), i.e., each is associated with that portion of the network closest to the respective arteriole, venule or FAZ. Specifically, we define the following seven ROI categories:

1. *Major radial arteriole*: ROIs are denoted “a1”, “a2”, … (shown in solid red in Fig. 2c)
2. *Capillary network around an arteriole*: ROIs are denoted “a1-net”, “a2-net”, … and do not include the arteriole (shown in outlined transparent red in Fig. 2c)
3. *Major radial venule*: ROIs are denoted “v1”, “v2”, … (shown in solid blue in Fig. 2c)
4. *Capillary network around a venule*: ROIs are denoted “v1-net”, “v2-net”, … and do not include the venule (shown in outlined transparent blue in Fig. 2c)
5. *Capillary network and small vessels around the foveal avascular zone*: single ROI denoted “F-net” (shown in transparent yellow in Fig. 2c) including the terminal capillary ring around the FAZ and some tiny arterioles (order “a-1”) and venules (order “v-1”)
6. *Band of pure capillaries around the foveal avascular zone*: single ROI denoted “F-cp” (shown outlined in solid yellow lines in Fig. 2c) including the terminal capillary ring and nearby capillaries
7. *Capillary network quadrant*: four ROIs denoted “Q1”, “Q2”, “Q3” and “Q4” that divide the macular area into four quadrants through the centre of the foveola, and exclude the major radial arterioles and venules ROIs and the FAZ (shown in outlined transparent green in Fig. 2d).

We note that the capillary network around the FAZ is particularly interesting. The foveola is roughly 350 µm across and is formed entirely by densely packed cones. The fovea is the next ring around the foveola. Capillaries appear in the inner retina and encircle the foveal slope, where they form the border of the FAZ. The role of capillary network around FAZ and their function are still not exactly known.

The definition of category 6 is informed by our existing knowledge about the capillaries around the FAZ based on our human donor eye studies^27^. Arguably the “F-cp” ROI is a unique location allowing us to gain reliable information concerning the CoV in these capillaries. This is because we can investigate the spatial and temporal variation of the capillaries without tiny arterioles and venules.

### ROI-based CoV analysis of the acquired OCTA data

Our custom software (“Image analysis” section) was used to perform CoV analysis of the OCTA data acquired for each subject. ROIs for the major radial arteriole and major radial venule categories, as well as for the FAZ, were defined interactively (using the suite of selection tools available in FIJI) whilst those for the other five “capillary network” categories were computed automatically by the software. Examples for each of the seven ROI categories are shown in Figs. 3a to 9a.

For each ROI, the software identified the individual vessel segments and for each segment computed the mean of its corresponding CoV values. The median and interquartile range (IQR) of these vessel-segment mean CoV values are measures of the spatial and temporal variation, respectively, of the vessel segments within the ROI.

For each subject and ROI category the spatio-temporal variation within each vessel was visualised by assigning a colour to the vessel based on its vessel-segment mean CoV value. Examples for each of the seven ROI categories are shown in Figs. 3b to 9b. Each plot has been rendered using a false colour map (a heat map from dark blue through to red). To accentuate differences in vessel-segment mean CoV value (differences in colour) between vessel segments, the colour bar scale has been clamped to the range of values defined by the smallest 25th percentile and largest 75th percentile of vessel-segment mean CoV values respectively across all the ROIs in the specific ROI category for that subject.

### Statistical analyses

Statistical analyses were performed using R with several additional packages. Notably, the *rstatix*^45^and *afex* packages^46^ were used to perform the repeated measures analysis of variance, and the *emmeans*^47^package was used to perform post-hoc multiple pairwise paired t-tests with *p* value correction. Tests were performed using a 5% level of significance.

## Supplementary Information

Supplementary Information 1.

Supplementary Information 2.

## Data Availability

The datasets generated during and/or analysed during the current study are available from the corresponding author on reasonable request.

## Code availability

The FIJI and R scripts can be found in the supplementary material online.
