# Supplementary material for: An assessment of microvascular hemodynamics in human macula
Source: Sci Rep. 2023 May 9;13:7550. doi: 10.1038/s41598-023-33490-8 (PMC10169832; doi:10.1038/s41598-023-33490-8)
Supplement: Supplementary file 2 — Supplementary Information 2. [file 41598_2023_33490_MOESM2_ESM.docx]

# **Supplementary tables**

Supplementary Table S1: Number of vessel segments contributing to each box plot in Fig. 10. Row number indicates subject.

|  | Arteriole | Arteriole-net | Venule | Venule-net | FAZ-net | FAZ-cp | Quadrant |
| --- | --- | --- | --- | --- | --- | --- | --- |
| 1 | 79 | 874 | 80 | 975 | 229 | 54 | 1951 |
| 2 | 45 | 1013 | 48 | 962 | 213 | 54 | 2076 |
| 3 | 78 | 963 | 65 | 849 | 160 | 66 | 1878 |
| 4 | 66 | 810 | 48 | 976 | 183 | 64 | 1867 |
| 5 | 50 | 1033 | 62 | 1014 | 166 | 45 | 2140 |
| 6 | 29 | 782 | 41 | 658 | 164 | 36 | 1553 |
| 7 | 70 | 934 | 54 | 642 | 145 | 45 | 1635 |
| 8 | 48 | 995 | 43 | 917 | 170 | 28 | 1996 |
| 9 | 45 | 828 | 44 | 761 | 262 | 59 | 1744 |
| 10 | 36 | 853 | 47 | 741 | 217 | 61 | 1727 |

Supplementary Table S2: Box plot medians (one for each ROI category) for each subject in Fig. 10. Row number indicates subject. Values reported to one decimal place.

|  | Arteriole | Arteriole-net | Venule | Venule-net | FAZ-net | FAZ-cp | Quadrant |
| --- | --- | --- | --- | --- | --- | --- | --- |
| 1 | 16.6 | 33.8 | 16.7 | 33.8 | 33.4 | 31.3 | 33.9 |
| 2 | 15.7 | 32.9 | 15.6 | 32.6 | 33.6 | 34.8 | 32.8 |
| 3 | 15.1 | 32.6 | 14.6 | 31.8 | 33.4 | 34.3 | 32.5 |
| 4 | 17.3 | 33.7 | 15.9 | 34.4 | 34.6 | 35.5 | 34.2 |
| 5 | 15.3 | 31.1 | 14.4 | 30.7 | 32.7 | 32.2 | 31.1 |
| 6 | 15.0 | 34.2 | 14.4 | 34.3 | 35.8 | 37.3 | 34.3 |
| 7 | 16.3 | 33.4 | 16.1 | 32.2 | 33.4 | 33.8 | 33.1 |
| 8 | 15.0 | 31.9 | 16.5 | 31.0 | 33.1 | 33.5 | 31.7 |
| 9 | 17.5 | 34.5 | 18.0 | 33.7 | 34.8 | 35.5 | 34.2 |
| 10 | 14.9 | 32.2 | 16.6 | 32.4 | 32.8 | 31.5 | 32.4 |

Supplementary Table S3: Box plot IQRs (one for each ROI category) for each subject in Fig. 10. Row number indicates subject. Values reported to one decimal place.

|  | Arteriole | Arteriole-net | Venule | Venule-net | FAZ-net | FAZ-cp | Quadrant |
| --- | --- | --- | --- | --- | --- | --- | --- |
| 1 | 4.9 | 11.8 | 6.3 | 10.5 | 10.8 | 10.2 | 10.8 |
| 2 | 3.1 | 9.9 | 4.0 | 9.9 | 10.3 | 12.5 | 9.7 |
| 3 | 5.0 | 10.8 | 3.5 | 9.5 | 11.2 | 10.5 | 10.1 |
| 4 | 6.5 | 10.0 | 4.9 | 11.1 | 11.1 | 8.7 | 10.5 |
| 5 | 3.5 | 9.4 | 4.1 | 10.1 | 10.6 | 8.6 | 9.6 |
| 6 | 3.2 | 9.6 | 3.4 | 9.1 | 10.3 | 10.5 | 9.4 |
| 7 | 5.6 | 9.8 | 4.4 | 9.4 | 8.9 | 7.6 | 9.6 |
| 8 | 4.3 | 9.8 | 5.2 | 10.6 | 8.7 | 8.2 | 9.9 |
| 9 | 3.6 | 12.5 | 4.9 | 11.0 | 11.5 | 8.5 | 11.6 |
| 10 | 4.9 | 11.1 | 5.4 | 10.8 | 10.1 | 10.1 | 10.5 |

Supplementary Table S4: Estimate, standard error and 95% confidence intervals for the difference in medians between each ROI category. The intervals have been adjusted based on the Bonferroni method. All values reported to two decimal places.

| contrast | estimate | SE | df | lower.CL | upper.CL |
| --- | --- | --- | --- | --- | --- |
| Arteriole - Arteriole.net | -17.18 | 0.27 | 9 | -18.32 | -16.04 |
| Arteriole - Venule | -0.01 | 0.31 | 9 | -1.33 | 1.30 |
| Arteriole - Venule.net | -16.84 | 0.34 | 9 | -18.28 | -15.40 |
| Arteriole - FAZ.net | -17.90 | 0.36 | 9 | -19.40 | -16.41 |
| Arteriole - FAZ.cp | -18.11 | 0.63 | 9 | -20.75 | -15.47 |
| Arteriole - Quadrant | -17.16 | 0.29 | 9 | -18.37 | -15.95 |
| Arteriole.net - Venule | 17.17 | 0.39 | 9 | 15.52 | 18.82 |
| Arteriole.net - Venule.net | 0.34 | 0.19 | 9 | -0.44 | 1.12 |
| Arteriole.net - FAZ.net | -0.72 | 0.21 | 9 | -1.60 | 0.16 |
| Arteriole.net - FAZ.cp | -0.93 | 0.50 | 9 | -3.03 | 1.17 |
| Arteriole.net - Quadrant | 0.02 | 0.08 | 9 | -0.32 | 0.36 |
| Venule - Venule.net | -16.83 | 0.48 | 9 | -18.84 | -14.83 |
| Venule - FAZ.net | -17.89 | 0.49 | 9 | -19.93 | -15.85 |
| Venule - FAZ.cp | -18.10 | 0.77 | 9 | -21.30 | -14.89 |
| Venule - Quadrant | -17.15 | 0.42 | 9 | -18.92 | -15.37 |
| Venule.net - FAZ.net | -1.06 | 0.25 | 9 | -2.11 | -0.01 |
| Venule.net - FAZ.cp | -1.27 | 0.54 | 9 | -3.53 | 1.00 |
| Venule.net - Quadrant | -0.32 | 0.11 | 9 | -0.78 | 0.15 |
| FAZ.net - FAZ.cp | -0.21 | 0.36 | 9 | -1.73 | 1.31 |
| FAZ.net - Quadrant | 0.74 | 0.20 | 9 | -0.10 | 1.59 |
| FAZ.cp - Quadrant | 0.95 | 0.50 | 9 | -1.15 | 3.05 |

Supplementary Table S5: Estimate, standard error and 95% confidence intervals for the difference in IQRs between each ROI category. The intervals have been adjusted based on the Bonferroni method. All values reported to two decimal places.

| contrast | estimate | SE | df | lower.CL | upper.CL |
| --- | --- | --- | --- | --- | --- |
| Arteriole - Arteriole.net | -6.01 | 0.47 | 9 | -7.98 | -4.04 |
| Arteriole - Venule | -0.16 | 0.36 | 9 | -1.66 | 1.35 |
| Arteriole - Venule.net | -5.75 | 0.36 | 9 | -7.25 | -4.25 |
| Arteriole - FAZ.net | -5.89 | 0.47 | 9 | -7.84 | -3.94 |
| Arteriole - FAZ.cp | -5.08 | 0.69 | 9 | -7.94 | -2.21 |
| Arteriole - Quadrant | -5.72 | 0.38 | 9 | -7.32 | -4.13 |
| Arteriole.net - Venule | 5.85 | 0.30 | 9 | 4.61 | 7.09 |
| Arteriole.net - Venule.net | 0.26 | 0.30 | 9 | -0.98 | 1.50 |
| Arteriole.net - FAZ.net | 0.12 | 0.30 | 9 | -1.14 | 1.38 |
| Arteriole.net - FAZ.cp | 0.93 | 0.55 | 9 | -1.38 | 3.25 |
| Arteriole.net - Quadrant | 0.28 | 0.16 | 9 | -0.37 | 0.94 |
| Venule - Venule.net | -5.59 | 0.19 | 9 | -6.39 | -4.79 |
| Venule - FAZ.net | -5.73 | 0.42 | 9 | -7.48 | -3.98 |
| Venule - FAZ.cp | -4.92 | 0.60 | 9 | -7.40 | -2.43 |
| Venule - Quadrant | -5.57 | 0.23 | 9 | -6.53 | -4.61 |
| Venule.net - FAZ.net | -0.14 | 0.32 | 9 | -1.47 | 1.20 |
| Venule.net - FAZ.cp | 0.67 | 0.56 | 9 | -1.65 | 3.00 |
| Venule.net - Quadrant | 0.02 | 0.16 | 9 | -0.63 | 0.68 |
| FAZ.net - FAZ.cp | 0.81 | 0.46 | 9 | -1.12 | 2.74 |
| FAZ.net - Quadrant | 0.16 | 0.24 | 9 | -0.85 | 1.17 |
| FAZ.cp - Quadrant | -0.65 | 0.54 | 9 | -2.89 | 1.59 |
